# Dataset on Brazilian Municipal Health Policies During the COVID-19 Pandemic

**DOI:** 10.1038/s41597-025-05870-4

**Published:** 2025-09-29

**Authors:** Andreza Aruska de Souza Santos, João Gabriel Ribeiro Pessanha Leal, Ester C. Sabino, Nuno R. Faria

**Affiliations:** 1 King’s Brazil Institute, School of Global Affairs, King’s College London, London, UK; 2 Escola de Saúde Pública Sergio Arouca, Fundação Oswaldo Cruz, Rio de Janeiro, Brazil; 3 Instituto de Medicina Tropical, Faculdade de Medicina da Universidade de São Paulo, São Paulo, Brazil; 4 MRC Centre for Global Infectious Disease Analysis, Department of Infectious Disease Epidemiology, School of Public Health, Imperial College London, London, UK

**Keywords:** Scientific community, Research data

## Abstract

Brazil was severely affected by COVID-19, in terms of both the number of infections and deaths. Part of the complexity and mismanagement of health measures in Brazil stemmed from the lack of nationwide policies, leaving local governments to play the key role in implementing pharmaceutical and non-pharmaceutical interventions. This fragmented response, with different health policies across 5,568 municipalities, posed significant challenges for researchers analysing dispersed data. This study consolidates information from 37 surveys conducted between 23 March 2021 and 24 March 2022 with mayors and health secretaries of Brazilian municipalities. The surveys, conducted by the Brazilian Confederation of Municipalities, achieved response rates ranging from 23.8% to 64.49%. Questions varied to address key epidemiological issues, including vaccination efforts, ICU bed availability, oxygen supplies, and restrictive measures. Disparities in response rates highlight Brazil’s regional and municipal size inequalities. This dataset, with 235 questions, provides essential insights into local governance, contributing to the study of outbreak management in contexts of inequality and political asymmetry.

## Background & Summary

Brazil was one of the countries most impacted by the COVID-19 pandemic in Latin America and the world in terms of the number of cases, deaths, and the duration of lockdowns^1,2^. Between 2020 and 2022, both pharmacological and non-pharmacological interventions (NPIs) were adopted at the municipal level, with 5,568 municipalities and the Federal District taking health-related action^3,4^. Here, we describe the complexity of asynchronous adoption and easing of pharmacological and non-pharmacological interventions in Brazilian municipalities.

Our data were gathered in a continuous municipal-level survey conducted by the Brazilian Confederation of Municipalities (Confederação Nacional de Municípios – CNM). This dataset adds to the results of a previously paper published during the first stage of the pandemic^5,6^, which discussed just one survey conducted between 13 May and 31 July 2020, rather than the 37 included in this dataset encompassing a broader period of time. The information offered now refers to local measures taken to address the pandemic in Brazil, and the dataset allows for a specific and detailed analysis of pharmacological and non-pharmacological policies that were implemented. In addition, it allows for future studies to measure the role of policies in the increase, spread, and duration of outbreaks.

### Data sources

Our dataset reveals the complexity of asynchronous and subnational health policies in Brazil by reporting data based on 37 surveys answered by mayors and health secretaries between 23 March 2021 and 24 March 2022.

The number of municipalities CNM interviewed in each survey varied over time. The database named *“Participation”* indicates in which round each municipality participated. The minimum number of participating municipalities for a round was 1,328 (23.8%), while the maximum reached 3,591 (64.49%), showing significant variation, while the median was 2,461 (44.19%) and the mean was 2,482 (44.57%).

**Fig. 1:**
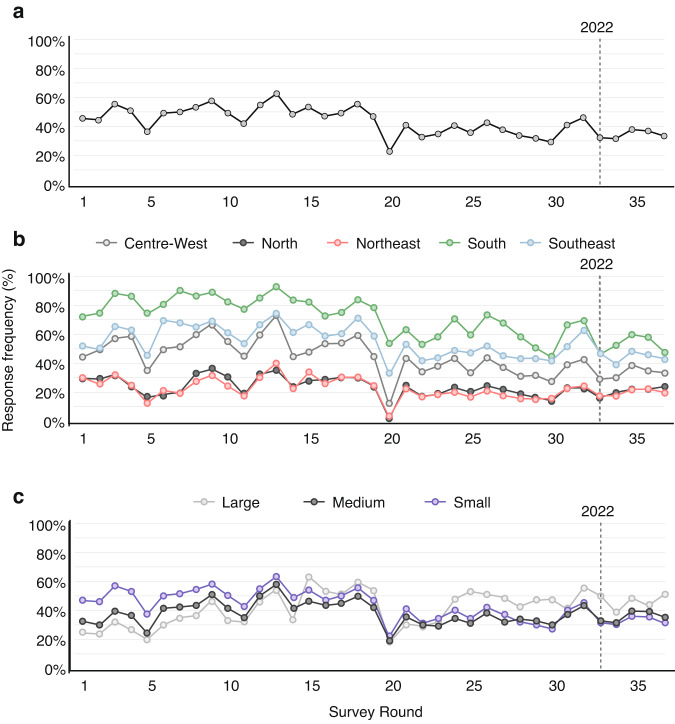
(**a**) Response rate (%) of municipalities, by survey round. (**b**) Response rate (%) of municipalities, by region and survey round. (**c**) Response rate (%) of municipalities, by municipality size and survey round.

Figure 1a shows the general response rate across all 37 survey rounds. Moments of greater response rate may be due to the relevance of the topic in that particular survey or the epidemiological moment of the pandemic. The numbers in 2022 reveal a light negative oscillation in the response rate.

Figure 1b shows the response rate in each different region, with significant regional differences. South and Southeast regions have higher response rates, frequently above 60%. North and Northeast regions present lower response rates, generally varying between 20% and 40%, while the Centre-West maintains an intermediary position. There were variations over time, however the tendencies described above were relatively stable.

Figure 1c categorises response rates according to the number of residents in each municipality (population size allows for the categorisation of large, medium, and small municipalities). Large municipalities (those with more than 300,000 inhabitants) show consistently high response rates, whereas small municipalities (those with up to 50,000 inhabitants) have a lower response rate, oscillating between 30% and 50%. Medium municipalities (those with a population between 50,000 and 300,000 inhabitants) present intermediary response rates, generally more aligned with the response rates of larger cities, but with inferior consistency. After March 2022 there is a slight reduction in the response rate across all municipal categories.

The above regional and city-size variation may have a two-fold explanation. First, historically CNM has a stronger presence in the South region of Brazil, and other surveys conducted by that organisation have shown higher response rates in that region where their presence and communication channels are stronger. Second, there are regional inequalities in Brazil with the North and the Northeast being poorer regions, which is reflected in local administrations that may have fewer staff members and may find it harder to answer surveys – particularly amid a health crisis.

### Contributions and recommendations

**Fig. 2:**
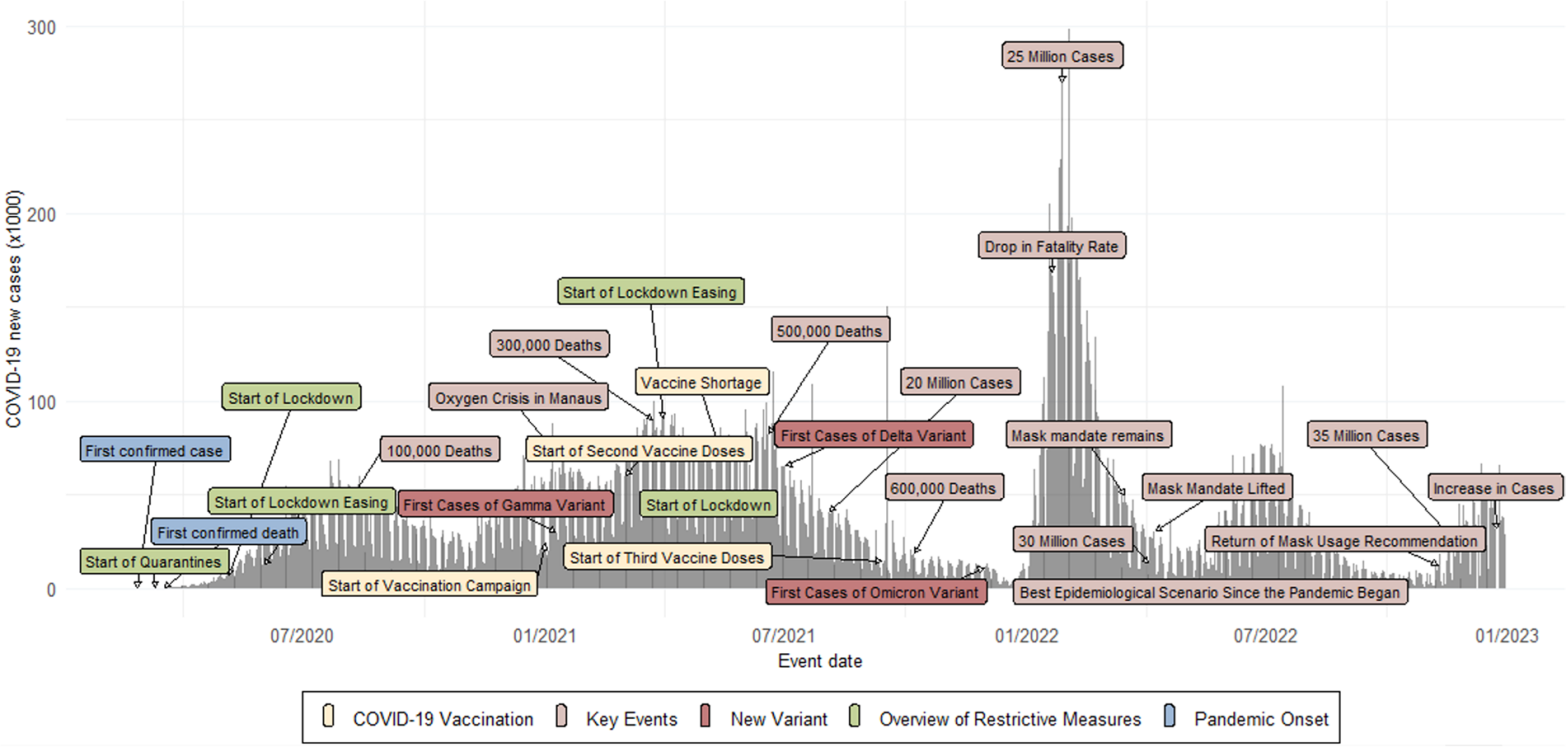
Timeline of significant events during the COVID-19 pandemic in Brazil.

As observed in Fig. 2, the pandemic in Brazil was marked by cycles of increasing and decreasing numbers of cases and deaths, which led to changes in policy needs and circumstances over time^7–9^. Brazil’s Supreme Court ruled that mayors and governors would have discretion in decisions related to the pandemic amid the absence of a national response to the pandemic, adding to the level of complexity in Brazil’s case^10^.

Brazil’s complex epidemiological data reflects a political moment under the presidency of Jair Bolsonaro, whose administration were against the adoption of pharmacological and non-pharmacological measures to control the pandemic, potentially worsening health results among the population^11–13^. The role of local policies grew in importance, making local data collection both urgent and difficult due to the large size of Brazil and its fragmented federation system.

This dataset meets the need to monitor and share information about fragmented policies designed to tackle health crises like the COVID-19 pandemic. Detailing these initiatives and how they varied across municipalities can help us to understand the effectiveness of interventions in reducing virus transmission. We offer information over time on a series of measures to encourage social distancing, implement the vaccination programme, provide infrastructure to treat infected people, and how local governments would eventually ease these measures. This information can be used by health systems in preparing for and assisting in future outbreaks where a highly contagious virus challenges society.

The evaluation of pandemic management policies in Brazil will need to consider the unequal duration of control measures, including non-pharmacological measures (physical distancing, isolation, quarantine, hand hygiene, and the use of face masks), environmental measures (surface cleaning and ventilation), and social measures (travel restrictions, school and workplace closures, and limits on gatherings) – as well as pharmacological measures (planning, distribution, evaluation, and oversight of the vaccination campaign to combat COVID-19) across the country. This dataset enables such evaluations, supporting future research and the development of public policies.

## Methods

### Data collection

Information on local NPI policies related to COVID-19 was collected through a telephone survey conducted directly with mayors, who had the option of receiving a password-protected link to respond to the online questionnaire later, update previous responses, or nominate health secretaries to respond. The dataset has three main pillars of information concerning pandemic policy response: the monitoring of restrictive measures, infrastructure to treat infected people, and the implementation of the vaccination programme. We have included the week that respondents received the questionnaire, the initial date when they started responding, and the last day for the respondent to submit their answers to the questionnaire.

Policy response to the pandemic, in this paper, refer to the set of actions undertaken by local authorities aiming to contain, mitigate, or manage the risks and consequences of COVID-19 within Brazilian municipalities. These actions go beyond routine healthcare services and encompass specific governmental decisions and interventions such as the adoption of restrictive measures (e.g., lockdowns, mask mandates), the expansion and adaptation of health infrastructure for the care of infected individuals, and the organization and execution of the local vaccination rollout.

We collaborated with the CNM to collect these data. The cooperation was formalised in a meeting with the CNM on 9 April 2020, and a written agreement was signed by the first and last authors of this article. The authors were given permission to describe, publish, and analyse the dataset. Prior to this current dataset with information from 2021 and 2022 across 37 surveys, the first and last authors had already shared an initial dataset on lockdown measures in Brazil that referred to a single survey and the paper was published on October 19 2020, in the early days of the pandemic^5^. The data we are now sharing are freely available to the public and to other academics for analysis. In this regard, this version stands out for its innovative approach of tracking, over the course of a year, the actions undertaken by mayors, providing a comprehensive databank of a wide range of public initiatives implemented by them.

As Brazil’s largest association of municipalities, the CNM has the email and phone numbers of all elected mayors in the country; the wide reach of the association makes it an ideal partner for sub-national data collection. The partnership was established to study the impact of decentralised measures in Brazil and the effects of decentralisation on the spread of infectious diseases.

### Data classification

The CNM conducted 37 rounds of questionnaires, totalling 235 questions. Questions can be browsed by theme. Questions can relate to the monitoring of restrictive measures where the main question is: “This week, the Municipality adopted/maintained any of the following measures”; questions that refer to the infrastructure needed to treat infected people, which include asking about the availability of intubation sets, hospital beds, and hospital oxygen. Another set of questions refers to the social consequences of the pandemic, and others focus on financial resources. Regarding the implementation of the vaccination programme, various questions address that topic, including questions on vaccination implementation programmes and completion time.

The percentage of municipalities that responded to the surveys varied over time. Figure 1 shows the response rate of the 5,586 Brazilian municipalities across each of the 37 survey rounds. Another aspect that varied was the duration of data collection for each survey. Data was collected between March 23, 2021, and March 24, 2022. The column *a_13_0* highlights the time interval required from the start to the end of each survey round. Additionally, since the topics covered during the pandemic also changed, the number of questions and the specific questions asked varied over time.

Therefore, in addition to the main database and its codebook, two auxiliary files are provided. The file named *“participation”* presents the response rate for each round, while the file named *“table_contention”* provides information on the number of times each question was asked throughout the surveys and the rounds in which it was included. These details will be explained further below.

## Data Records

This dataset gathers information on the processes and activities of the pandemic response in Brazilian municipalities, and has been deposited in Dryad, a platform dedicated to hosting and sharing academic datasets^14^.

Four documents are available: the database (*bank_measures_complete.Rda*), the codebook (*codebook_complete.csv*), the table with the municipal participation rate (*participation.csv*), and a table detailing which questions are included in each round of the surveys (*table_contention.csv*)^14^. Further details about each document are provided below.

### Main dataset: bank_measures_complete

This is the database containing all the questions and all the rounds conducted with the municipalities. The document *codebook_complete.csv* provides the codebook for all the variables present in the database.

### Livro de Códigos: codebook_complete

The codebook provides a detailed description of the information and the explanations of the respective codes present in the *bank_measures_complete.Rda* database.

Detailed information in the file *codebook_complete.csv*:

- *group*: The “group” variable gathers a set of questions related to the same analytical dimension.
- *letter*: For each “group”, that is, for each dimension that groups a series of questions, a letter from the alphabet is assigned as a code.
- *code*: This variable indicates the number that the corresponding information represents in the *bank_measures_complete.Rda* database.
- *questions*: This presents the description of the question asked, which is recorded in the *bank_measures_complete.Rda* database.
- *variables*: This variable indicates the code of the corresponding question in the *bank_measures_complete.Rda* database.
- *values*: “Values” provides the explanations for each coding associated with the values assigned to a given response.

### Support File 1: participation

This table presents the percentages of municipalities that participated in each round of interviews, considering the total number of municipalities in Brazil.

Detailed information in the file *participation*:

- *interview sequence:* In this variable, the order of interviews is presented in ascending order, ranging from 1 to 37.
- *interview week:* The date on which the interviews were conducted.
- *responding municipalities:* The number of municipalities that participated in the mentioned round of interviews.
- *total municipalities:* Total number of municipalities in Brazil.
- *% of respondents:* The percentage of municipalities that participated in each round of interviews relative to the total number of municipalities.

### Support File 2: table_contention

The document provides the total number of occurrences of each question throughout the 37 rounds and lists the rounds in which this question appeared. Over time, different questions were asked to the mayors that reflect different stages of the pandemic, and the table in question shows the frequency of each one, as well as indicating in which specific rounds they were conducted.

Detailed information in the file *table_contention.csv*:

- *group*: The “group” variable gathers a set of questions related to the same analytical dimension.
- *letter*: For each “group”, that is, for each dimension that groups a series of questions, a letter from the alphabet is assigned as a code.
- *code*: This variable indicates the number that the corresponding information represents in the *bank_measures_complete.Rda* database.
- *questions*: This presents the description of the question asked, which is recorded in the *bank_measures_complete.Rda* database.
- *variables*: This variable indicates the code of the corresponding question in the *bank_measures_complete.Rda* database.
- *how_many_times_it_appears*: The number of times a specific question was asked across the 37 rounds of interviews. Thus, a question could have been asked once or up to 37 times, corresponding to the total number of rounds conducted.

The remaining variables indicate whether a specific question is included in a particular round of the survey. “Yes” indicates that the question is present in this specific round, while “No” means that the question was not asked in this round.

## Usage Notes

We invite researchers to use these data to deepen their understanding of the pandemic and support health policymakers’ efforts in other health emergencies.

Additionally, by combining our dataset with other government sources, such as those from the Brazilian Superior Electoral Court, we offer tools to investigate the effects of politics on public health policies during the pandemic and thus generate institutional learning for health systems, empirically demonstrating how political decisions influence public health policies. For example, we can analyse how the alignment of a particular mayor with the former president, elected in 2018, their party affiliation, and other such factors affected political decisions relating to pandemic response measures.

## Technical Validation

Interviewing mayors and health secretaries provides an important perspective for interpreting laws and understanding the start and end of pharmacological and non-pharmacological interventions. While decrees restricting physical contact or easing social distancing measures are available online, there are often multiple laws addressing similar issues (e.g., a decree closing non-essential services, followed by another defining what constitutes non-essential services, and a third determining the duration of the closure).

From the perspective of pharmacological actions, there are instances where the allocation strategies for interventions, as well as specific issues, varied at the local level. Notably, based on available data, it is possible to closely monitor the evolution of vaccination campaigns in municipalities, such as the population’s reception of a specific vaccine, vaccine shortages, and other situations better understood and reported through information collected from mayors and secretaries – policymakers embedded in the local context.

Our data validation process included the broad dissemination of information. The CNM periodically published reports summarising the questions and answers from each round of the survey. These reports are publicly available and can be accessed via the following link^15^: https://cnm.org.br/biblioteca?search=Pesquisa%20CNM%20-%20Covid-19.

The identification of the survey round on the website can be done in two ways: by the numbering corresponding to the data entered in column *a_13* of the main database *“bank_measures_complete”*, or by the survey date recorded in column *a_13_0*.

To access the reports, follow these steps:

1. Go to the library page on the CNM website.
2. In the advanced search area, type “Pesquisa CNM – COVID-19”.
3. This will provide access to all the available information.

These publications served as a validation of the database in two ways: (1) they allowed respondents to dispute the results or question the methods if necessary; (2) they disseminated the results to a broad audience, enabling interaction with their municipalities and/or the CNM. So far, no disputes regarding the data or refusals to disclose it have been reported by respondents or other users of the CNM platform.

Additionally, due to the data collection process, some erroneous entries may have occurred in specific cases, such as dates for easing NPIs being the same as or earlier than the dates of implementing social distancing measures. In aggregated analyses, these errors should not pose a significant issue. However, for studies focused on specific municipalities, we recommend consulting other information sources, such as the municipality’s official gazette, for complementary analyses.

As not all municipal authorities answered all the questions, we suggest that users of this dataset consider additional sources of information to document the implementation of missing policies, preferably using official sources of information such as local decrees. However, as decrees are not always available online, secondary sources, such as media reports, may need to be consulted.

In the dataset describing the 2020 survey^6^, observations obtained from the CNM on the adoption of NPIs were compared with data collected through municipal decrees in capital cities. This comparison included eight cities featured in both the CNM interviews and the compilation of decrees conducted by the Oxford COVID-19 government response tracker (www.bsg.ox.ac.uk/research/covid-19-government-response-tracker)^16^. At the time, it was found that, when comparing the information gathered by the Oxford team from the decrees with that obtained through the interviews, there were no discrepancies. This validation is important because, given the impracticality of analysing numerous decrees from over 5,000 cities, the survey becomes essential, and the reliability of its data is paramount.

## Data Availability

To achieve the encoded and prepared database ready for use, a series of modifications were carried out. The script demonstrating the actions taken can be found on Dryad 10.5061/dryad.v6wwpzh5h and in the document called ‘manipulation_base’. The modifications were performed using the software R Studio, version 4.3.1. The aforementioned script presents the code used to assemble the following files: bank_measures_complete, participation, and table_contention. Additionally, the script used to conduct the analyses presented in this paper is also available and can be accessed through the document ‘script_nature’.
